# Long‐Term Graft Survival and Visual Outcomes in Deep Anterior Lamellar Keratoplasty (DALK): A Narrative Review

**DOI:** 10.1155/joph/8120487

**Published:** 2026-02-23

**Authors:** Sadegh Ghafarian, Ellen H. Koo

**Affiliations:** ^1^ Department of Ophthalmology, School of Medicine, Farabi Eye Hospital, Tehran University of Medical Sciences, Tehran, Iran, tums.ac.ir; ^2^ Department of Ophthalmology, Bascom Palmer Eye Institutes, University of Miami Miller School of Medicine, Miami, Florida, USA, miami.edu

## Abstract

Deep anterior lamellar keratoplasty (DALK) selectively replaces the scarred or diseased corneal stroma, while preserving healthy endothelium, thus providing an alternative to penetrating keratoplasty (PK) for patients with stromal disorders. Despite its recognized advantages, DALK has not become as widely adopted as anticipated over the past decades. The satisfactory visual outcomes of PK, the lack of standardized DALK techniques, as well as the limited long‐term data on visual and refractive outcomes for both DALK and PK are commonly cited as barriers to its broader implementation. Evidence from long‐term studies indicates that DALK offers superior graft survival and provides a safer intraoperative profile with fewer complications and lower endothelial cell loss, making it the preferred option for stromal diseases with healthy endothelium.

## 1. Introduction

For over half a century, penetrating keratoplasty (PK), involving the full‐thickness replacement of diseased corneal tissue with a donor graft, has been a cornerstone in the surgical management of various corneal diseases [1, 2]. Despite its success, PK carries a lifelong risk of endothelial rejection and graft failure, with progressive endothelial cell loss persisting for years after surgery [3]. Additionally, the full‐thickness nature of the technique puts the recipient at risk for traumatic graft dehiscence. These limitations have driven the development of selective lamellar transplantation techniques, which offer a safer, more targeted treatment approach.

Deep anterior lamellar keratoplasty (DALK) has emerged as a preferred alternative to PK for treating disorders primarily involving the corneal stroma with intact endothelium, such as keratoconus, stromal scars, and stromal corneal dystrophies [4]. By preserving the recipient’s endothelium, DALK offers several advantages, including a reduced risk of endothelial rejection, a lower incidence of secondary glaucoma, and a more favorable biomechanical safety profile in the event of trauma [5]. The closed‐system nature of the surgery confers intraoperative advantages of circumventing the risks associated with open‐sky surgery as seen with PK.

The refinement of DALK, particularly through the introduction of techniques such as the big‐bubble (BB) method by Anwar and Teichman [6], and more recently, femtosecond laser–assisted DALK (Femto‐DALK), has further improved its safety and efficacy, making it a suitable option for a broader range of patients. Although the benefits of DALK are well documented in the literature, long‐term outcomes related to graft survival, visual acuity, and refractive results remain varied and inconsistent [7, 8]. These outcomes are shaped by numerous factors, including indication for surgery, surgical techniques, patient‐specific characteristics, and postoperative management strategies [9].

This review focuses on the long‐term graft survival and visual outcomes of DALK, with a particular emphasis on its immunologic advantages in comparison to PK. While previous studies have provided valuable insights, long‐term data remain limited and fragmented, leaving uncertainty regarding the optimal clinical approach. By exploring and critically appraising the emerging long‐term evidence and highlighting new advancements, this narrative review aims to clarify the comparative outcomes, immunologic differences, and complication profiles of DALK versus PK, providing clinicians and researchers with a comprehensive resource to guide evidence‐based management of various corneal disorders.

## 2. Methods

### 2.1. Literature Search Strategy

A comprehensive literature search was performed to identify studies evaluating long‐term graft survival and visual outcomes following DALK and PK. The electronic databases PubMed/MEDLINE, Embase, Scopus, and Web of Science were searched for articles published up to May 2025. Search terms included combinations of the following keywords and Medical Subject Headings (MeSH): “Deep Anterior Lamellar Keratoplasty” OR “DALK”, “Penetrating Keratoplasty” OR “PKP” OR “PK” OR “Full‐thickness Keratoplasty”, and “long‐term outcomes” OR “graft survival” OR “graft failure” OR “visual acuity” OR “visual outcomes” OR “follow‐up” OR “prognosis” OR “complications” OR “endothelial cell density”. Boolean operators were applied to combine terms. Reference lists of relevant articles and review papers were also manually screened to identify additional eligible studies.

### 2.2. Eligibility Criteria

Studies were included if they met the following criteria: (1) original clinical research (prospective, retrospective, case series, or clinical trials); (2) a minimum follow‐up duration of 5 years; (3) reporting outcomes on graft survival, corrected distance visual acuity (CDVA), or long‐term complications following DALK or PK; and (4) published in English. Exclusion criteria were review articles, editorials, expert opinions, conference abstracts without primary data, single case reports, and studies with fewer than 10 patients.

### 2.3. Study Selection and Data Extraction

Titles and abstracts were screened to identify potentially relevant studies, and full texts were retrieved for detailed evaluation. From each eligible study, the following data were extracted: study design, indication for keratoplasty, surgical technique, sample size, duration of follow‐up, definitions and rates of graft survival, CDVA, and complications (e.g., graft rejection, interface haze, secondary interventions, or conversion to PK in DALK cases).

## 3. Immunologic Aspects of Keratoplasty

### 3.1. Immune Privilege in the Corneal Transplantation

The first successful corneal transplant was performed in 1905 by Austrian ophthalmologist Eduard Zirm [10] and has become popular since then. Its exceptionally high success rate compared to other tissue transplants is largely attributed to the unique phenomenon of “immune privilege” in the cornea [11], which is driven by three major mechanisms:

1. Anatomical, Cellular, and Molecular Barriers: The cornea’s avascular nature (absence of both lymphatic and blood vessels) plays a critical role in reducing immune cell infiltration and antigen presentation that is regulated by limbal epithelial cells [12]. Additionally, corneal endothelial cells exhibit low expression of major histocompatibility complex (MHC) class I molecules, which reduce T‐cell recognition and facilitate the apoptosis of CD95+ T cells through the CD95 ligand (FasL) pathway [13, 14].
2. Immunosuppressive Intraocular Microenvironment: The aqueous humor contains soluble immunomodulatory factors secreted by the ciliary body, including α‐melanocyte‐stimulating hormone (α‐MSH) and vasoactive intestinal peptide (VIP). These factors suppress inflammatory responses [15]. The cornea, iris pigment epithelium (IPE), and retinal pigment epithelium (RPE) produce immunosuppressive molecules such as transforming growth factor‐β (TGF‐β) and thrombospondins‐1 and ‐2 (TSP‐1/TSP‐2). These molecules modulate T‐cell function, reducing their capacity to initiate immune responses [16, 17].
3. Immune Tolerance Mechanisms: The anterior chamber‐associated immune deviation (ACAID) is a form of systemic immune tolerance to antigens introduced into the anterior chamber, which selectively suppresses antigen‐specific delayed‐type hypersensitivity (DTH) responses through downregulation of CD4^+^ Th1 and Th2 cells, while promoting humoral immunity and the production of non‐complement‐fixing antibodies. Regulatory T cells (Tregs) are crucial in maintaining this tolerance and preventing graft rejection [18, 19].

Despite these protective mechanisms, immune‐mediated corneal allograft rejection remains a significant concern, particularly after PK, with endothelial rejection being the main cause of graft failure, accounting for approximately 28% of cases [20]. Endothelial failure often develops following an episode of endothelial rejection due to the substantial loss of endothelial cells during the rejection process [21]. By preserving the recipient’s endothelium, DALK eliminates the risk of endothelial rejection, thereby significantly reducing the likelihood of immune rejection and subsequent late graft failure. Moreover, DALK offers additional immunologic advantages, including a reduced antigen load due to the selective transplantation of stromal tissue and the potential immunomodulatory benefits of ACAID [22].

### 3.2. Post‐DALK Immune Reactions

Allograft rejection remains the primary cause of graft failure in corneal transplantation. Over a 10‐year period, approximately 19% of low‐risk PK grafts experience at least one rejection episode, while the incidence rises to 30%–60% for high‐risk PK grafts [23, 24]. Furthermore, a recent prospective cohort from the Singapore Corneal Transplant Study found that irreversible allograft rejection and late endothelial failure collectively accounted for over 60% of PK graft failures over a 20‐year period [25]. Since the recipient’s Descemet membrane (DM) and endothelium are preserved after DALK, endothelial immune reactions would be prevented. Consequently, only epithelial, subepithelial, or stromal immune responses—similar to those seen in PK rejection—can develop. Stromal rejection is the primary immune reaction after DALK, presenting as diffuse or sectoral graft stromal infiltrates, edema, and vascularization extending across the donor–recipient junction or interface, which, if left untreated, can lead to graft opacification [26, 27].

A recent systematic review, which included 530 eyes that underwent PK and 568 eyes that underwent DALK from 13 studies, concluded that the risk of graft rejection episodes was significantly higher in the PK group, with a rejection rate of 21.3%, compared to 8.95% in the DALK group [5]. Long‐term studies (> 5 years) report that the rejection rate for DALK ranges between 0% and 19.7% [28, 29]. While most studies evaluating long‐term graft survival in DALK and PK indicate that rejection is less likely to occur following DALK compared to PK [7, 25, 30], some studies have reported no significant differences [24].

Although DALK eliminates the risk of endothelial graft rejection, surprisingly, the likelihood of developing subepithelial and stromal immune reactions appears to be significantly higher compared to PK [31, 32]. Janiszewska‐Bil et al. observed subepithelial opacifications, indicative of subepithelial rejection, in 26% of DALK patients. These cases responded well to intensive treatment, with no graft failures reported as a result of rejection [33]. An AAO report [34] evaluated the long‐term outcomes of 142 DALK procedures compared to an equal number of matched PK procedures in patients with various corneal diseases. Subepithelial and stromal rejection occurred in 12% of DALK cases over a follow‐up period of 42.9 ± 22.9 months, compared to just 2% in the PK group over 80.5 ± 50.3 months. Similarly, Feizi et al. reported a 19.7% incidence of subepithelial and stromal rejection in patients undergoing DALK for advanced keratoconus over a follow‐up period of 72.9 ± 47.8 months [31]. Gonzalez et al. also reported a 14% risk of stromal rejection over 18 months in a cohort of 251 DALK procedures [32]. This higher incidence of subepithelial and stromal immune reactions after DALK could be explained by the fact that, in DALK, donor antigens are not introduced into the anterior chamber, which prevents the activation of ACAID mechanisms [27].

In a comparison of two common techniques for DALK (BB vs. Manual dissection), there was no difference in the rate of immune failure between the DALK groups, underscoring the crucial role of host endothelium preservation in preventing immune failure [24].

While nonendothelial immune reactions, primarily stromal, may still occur, these are generally well managed with frequent topical corticosteroids, leading to favorable vision or graft outcomes [31, 32]. In contrast, endothelial rejection in PK often leads to graft failure [35, 36]. Furthermore, the risk of epithelial, subepithelial, and stromal rejection episodes appears to diminish over time as host epithelial cells and keratocytes repopulate the donor tissue [27, 37]. Nonetheless, late rejection episodes, occurring 3–4 years postsurgery, have also been reported [38]. It is important to note that while the risk of rejection episodes in DALK appears to decrease over time, rejection can occur throughout the lifespan of the graft, and the risk is not confined to the early postoperative years [39].

### 3.3. Advances in Managing Immune Responses

Topical corticosteroids are widely regarded as the cornerstone of immunosuppressive therapy following corneal transplantation. They effectively inhibit both corneal hemangiogenesis and lymphangiogenesis, thereby reducing the risk of immune‐mediated graft rejection [40]. While systemic immunosuppressive agents are typically unnecessary in most cases, their use in high‐risk transplants remains a subject of debate due to the lack of a consensus [41]. One proposed advantage of DALK over PK is the possibility of a shorter corticosteroid regimen. However, subsequent studies have revealed significant rates of stromal rejection following DALK, particularly where a relatively brief topical corticosteroid regimen is employed [42, 43]. Gonzalez et al. reported a 14% risk of stromal rejection within the first postoperative year, a rate notably higher than those observed in previous studies. The authors attributed this increased risk to the use of a relatively short postoperative corticosteroid regimen, with a median duration of only 7 weeks [32]. This suggests that the corneal stroma retains immunogenic properties capable of eliciting immune‐mediated graft reactions. Accordingly, a topical corticosteroid regimen extending up to 1 year, similar to that used in PK, is recommended to minimize the risk of stromal rejection following DALK [26, 43]. However, given the potential adverse effects, the risks and benefits of long‐term steroid therapy should be carefully assessed for each patient.

Episodes of corneal allograft rejection are typically treated with frequent administration of corticosteroid eye drops, such as prednisolone acetate 1%, dexamethasone 0.1%, or difluprednate 0.05%, with systemic corticosteroids rarely required. These medications are gradually tapered over several weeks as symptoms resolve. Timely recognition and aggressive management are crucial for achieving optimal visual and anatomic outcomes [44, 45]. Although the effectiveness of topical cyclosporine A (0.05%–2%) remains unclear, topical tacrolimus (typically 0.03%) has shown promise in managing high‐risk corneal transplantation cases [46, 47]. Systemic agents such as cyclosporine A, mycophenolate mofetil, tacrolimus, sirolimus, and rapamycin have all been explored for corneal graft rejection. However, these regimens exhibit variable success rates, carry significant systemic side effects, and require individualized planning along with close monitoring [23, 48].

Recent advancements, such as engineered controlled‐release nanomedicine formulations combining immunosuppressants and antiangiogenic agents have shown promise in preventing high‐risk graft rejection in animal models while enhancing ocular drug delivery and reducing toxicity and dosing frequency [49]. Additionally, long‐term cryopreserved corneal tissues have been shown to be safe and effective for use in DALK, providing clinical outcomes comparable to those achieved with fresh corneal tissues [50, 51]. These preserved tissues help expand the available donor pool by allowing the use of corneas from donors lacking endothelial viability, tissues that would otherwise be unsuitable for PK. Moreover, the absence of viable cells, including keratocytes and antigen‐presenting cells, is believed to reduce the risk of allograft rejection following DALK [50–52]. In this context, gamma‐irradiated sterile cornea (GISC) represents a further advancement in corneal graft technology [53]. GISC is particularly advantageous in procedures where a functional endothelium is not required—such as DALK and glaucoma patch grafts [54, 55]. Its application is associated with a significantly lower risk of immunologic rejection and infection, attributable to the sterilizing and antigen‐lowering effects of gamma irradiation [56].

## 4. Long‐Term Graft Survival and Visual Outcomes

### 4.1. Graft Survival

Graft survival depends on multiple factors, including episodes of rejection, postoperative complications, and endothelial cell density (ECD). ECD is the most important factor, serving as a proxy for graft survival and ensuring graft clarity. Endothelial rejection and the associated endothelial cell loss are the most commonly reported causes of corneal graft failure after PK, accounting for 25% of all failed grafts [57]. Even in the absence of rejection, substantial endothelial cell loss occurs following PK. The Cornea Donor Study reported a 76% reduction in ECD from baseline 10 years after PK, with only 14% of patients retaining an ECD > 1000 cells/mm^2^ [58]. While PK graft survival was favorable in the short term (94.4% at 1 year postoperatively), its long‐term survival declined to 72% at 10 years [59], with endothelial cell loss being most pronounced in the first year (43%) and continuing at a rate of approximately 33% at two years, 54% at three years, and 59%–70% at five years—far exceeding the normal physiological endothelial cell loss rate of 0.6% per year [60–62]. Australian Corneal Graft Registry further demonstrated a progressive decline in PK graft survival for keratoconus, from 89% at 10 years to 49% at 20 years, and just 17% at 23 years, highlighting the impact of progressive endothelial cell loss over time [63].

We evaluated studies on long‐term graft survival after DALK; given the heterogeneity of studies, we have summarized original articles evaluating graft survival for ≥ 5 years, with or without comparison to PK, that included a sufficient number of cases, in Table 1. Most studies have demonstrated a higher graft survival rate after DALK compared to PK [24, 29, 34, 59]. In a few studies, graft survival was comparable between DALK and PK; however, DALK still showed superior outcomes with lower complication rates, lower EC loss, and fewer rejection episodes [31, 63]. Borderie et al. reported the longest follow‐up duration in the literature for DALK and PK outcomes in keratoconus, with an average of 9 years and a maximum of 27 years. They found that the 20‐year graft survival rates were comparable, with 94.9% for PK and 97.0% for DALK [24]. However, these survival rates were higher than those reported in other studies, likely due to the study’s monocentric design with prospective patient follow‐up, which minimized the impact of the surgeon’s learning curve and facilitated early diagnosis and management of postoperative complications.

**TABLE 1:** Summary of major original studies evaluating long‐term surgical outcomes after DALK.

Study	Indication	Sample size (no. of eyes)	Mean follow‐up ± SD (m)	Graft survival rate (%) at the last follow‐up	ECD (cells/mm^2^) or ECL (%)	Graft rejection episode (%)	Main finding
Arundhati et al. (2020) [59]	Various conditions	DALK 362 PK 307	40.0 ± 38.9	DALK 93.9% at 5 y 93.9% at 10 y PK 80.4% at 5 y 72.0% at 10 y	Not assessed	DALK 1.7% PK 16.6%	The 10‐year graft survival rate for DALK was superior to that of PK, with fewer postoperative complications and lower rates of graft rejection and failure

Borderie et al. (2024) [24]	Keratoconus	DALK 228 PK 274	DALK 103.4 PK 106.1	DALK 97.1% at 15 y PK 96.4% at 15 y	Mean ECD at 10 y DALK 1966 PK 827	DALK 16.7% PK 19%	DALK features higher long‐term endothelial survival and a lower risk of postoperative ocular hypertension compared with PK

Borderie et al. (2012) [34]	Various conditions	DALK 142 PK 142	DALK 42.9 ± 22.9 PK 80.5 ± 50.3	Not mentioned	ECL DALK −22.3% PK −50.1%	DALK 12% PK 22%	In the long term, model‐predicted graft survival and endothelial density are higher after DALK than after PK

Feizi et al. (2022) [31]	Keratoconus	DALK 193 PK 218	DALK 72.9 ± 47.8 PK 77.9 ± 46.5	DALK 94.8% PK 98.2%	Not assessed	DALK 19.5% PK 33.5%	PK was associated with a higher rate of graft rejection. Both techniques were comparable in terms of graft survival

Kobaloghlu et al. (2011) [61]	Keratoconus	DALK 234	50.5 ± 22.2	Not mentioned	ECL at 8 years −22.5% ± 15.9%	1.7% stromal	DALK exhibits lower long‐term endothelial cell loss compared to PK

MacIntyre et al. (2014) [63]	Keratoconus	DALK 31 PK 42	DALK 51.8 PK 53.7	DALK 93% PK 100%	Not assessed	DALK 3.2% PK 16.7%	DALK showed similar graft survival and fewer postoperative complications compared to PK

Ogawa et al. (2016) [62]	Various conditions	DALK 275	51 ± 41	83.5% at 5 y 74.1% at 10 y	Mean ECD was > 1000 at 5 y	2.2.%	The graft survival rate of DALK was favorable, with low rejection rates and a slight decrease in ECD over the long term

Romano et al. (2015) [37]	Keratoconus	DALK 158	76.9 ± 23.2	Not mentioned	Mean ECD at last F/U 2070.5 ± 367.5	11.3% Stromal, epithelial	The risk of graft rejection, postoperative complications, and late endothelial cell loss is lower compared to standard PK

Zhang et al. (2013) [29]	Keratoconus	DALK 75 PK 52	DALK 46.9 ± 28.0 PK 60.2 ± 34.6	DALK 100% PK 98.1%	ECL at 5 y DALK −13.9% PK −34.6%	DALK 0% PK 7.7%	Graft rejection, secondary glaucoma, and continuous endothelial cell loss were observed exclusively after PK

*Note:* m, month; y, year.

Abbreviations: DALK, deep anterior lamellar keratoplasty; ECD, endothelial cell density; ECL, endothelial cell loss; F/U, follow‐up; PK, penetrating keratoplasty; SD, standard deviation.

Current literature shows considerable variability in graft survival outcomes, with 10‐year survival rates ranging from 74.1% to 97.1% for DALK and from 72% to 96.4% for PK [24, 59, 62]. This variability arises from multiple factors, including differences in surgery indications and nonuniform exclusion criteria. For instance, studies with a higher proportion of keratoconus cases tend to report better survival rates [31], whereas those with a greater percentage of high‐risk grafts report lower survival rates [62]. Even in a study reporting high graft survival rates of up to 97%, the mean corneal ECD at the 10‐year follow‐up was 1966 cells/mm^2^ in DALK and 827 cells/mm^2^ in PK. They also observed that both early‐ and late‐phase endothelial cell loss rates were significantly higher in PK than in DALK, with PK exhibiting a higher CCT than DALK at the last examination [24].

Almost all studies have reported higher long‐term endothelial cell survival after DALK compared to PK (Table 1), with endothelial cell loss being nearly twice as high in PK compared to DALK [29, 34]. Based on these findings, some authors applied a joint regression model to predict very long‐term graft survival. Using this model, the predicted 50‐year graft survival was significantly higher for DALK compared to PK, with a median model‐predicted graft survival of 49 years in the DALK group versus 17.3 years in the PK group [24, 34]. Since patients with keratoconus are relatively young and often undergo keratoplasty at an earlier age, long‐term survival and function of the corneal endothelium are crucial for maintaining their lifelong visual abilities.

### 4.2. Visual Outcomes

Studies on the long‐term (5‐year) outcomes of DALK and PK have concluded that both techniques significantly improve visual and refractive outcomes, with no significant difference between them [24, 63]. DALK and PK also yield comparable results in terms of contrast sensitivity and higher‐order aberrations (HOAs) [64–66]. A recent meta‐analysis by Awad et al., which included 12 studies assessing CDVA at 2 years, found no significant difference between PK and DALK [66]. The results of another meta‐analysis by Shams et al., which included 18 studies, also found an insignificant 1% improvement in CDVA in the DALK group as compared with the PK group [8]. Conversely, some studies have reported better visual outcomes with PK. Feizi et al., in a study with a 6‐year follow‐up on surgical management of advanced keratoconus, found significantly better postoperative UDVA and CDVA in the PK group, with CDVA at 0.18 ± 0.13 logMAR versus 0.26 ± 0.19 logMAR in DALK [31].

Understanding these conflicting results requires considering key factors. The term “DALK” encompasses both manual dissection, which may leave residual stromal tissue, and BB DALK, which fully exposes DM. Studies suggest manual dissection results in inferior CDVA and slower visual recovery compared to PK and BB DALK, while BB DALK shows no difference from PK [24, 34]. Romano et al. observed that keratoconus eyes with BB DALK had better CDVA than those with manual dissection at follow‐up less than 5 years, but interestingly, long‐term outcomes were comparable [37]. The main factor influencing visual outcomes after DALK appears to be the thickness of the residual recipient stromal bed. Ardjomand et al. demonstrated that a residual bed < 20 μm achieves visual results comparable to PK, while a thickness > 80 μm significantly reduces visual acuity [64], a finding further confirmed by Alió et al. [67]. Similarly, Reinhart et al. concluded that a residual stromal bed exceeding 10% of total stromal thickness is associated with lower visual acuity [2].

Additionally, surgical indications influence the visual outcomes. Ogawa et al. found that at 5 years post‐DALK, CDVA was significantly better in the keratoconus group than in the dystrophy group [62]. Even among keratoconus patients undergoing DALK, those with advanced disease (central curvature > 60 D) are more likely to develop DM folds in the visual axis, which can adversely affect visual acuity. These folds result from a mismatch between the redundant recipient DM and the posterior surface of the donor graft [68]. Visible interface folds were present in 17% of DALK in advanced cases early postoperatively, resolving in most cases over time and after suture removal [31]. However, even if clinically undetectable, subtle microfolds can persist long‐term and have been identified using confocal microscopy up to 2 years postoperatively [69].

Overall, when optimal surgical technique is achieved, particularly BB DALK with a thin residual stromal bed, long‐term visual outcomes are comparable to those of PK.

### 4.3. Refractive Outcomes

While some studies including the Australian registry study found no significant difference between DALK and PK in spherical equivalent or astigmatic refractive outcomes [63, 70], others reported better results with PK [31, 71]. Despite significant heterogeneity among studies, a recent meta‐analysis found that at 2 years postoperatively, PK was associated with a lower spherical equivalent, lower mean corneal curvature, and lower topographic corneal power compared to DALK, while corneal astigmatism showed no significant difference [66]. Huang et al. reported refractive outcomes of keratoplasty for keratoconus at a 9‐year follow‐up, finding a mean myopic refractive sphere of −5.0 ± 1.5 D in the PK group and −6.1 ± 1.8 D in the DALK group [71]. While this difference was statistically significant, a 1.0 D difference may not be clinically relevant, especially given the comparable CDVA between groups.

The refractive outcome of corneal transplantation is influenced by factors such as trephination size, donor tissue distribution, suturing technique, and graft–host junction healing [72–74]. Therefore, a clinically significant difference in astigmatism or refractive error between DALK and PK is not expected. Refraction usually stabilizes 6 months after complete DALK suture removal, after which additional refractive correction procedures can be considered [24, 62, 75].

## 5. Complications and Their Impact on Long‐Term Outcomes

Although PK is effective in managing most stromal corneal pathologies and shows comparable outcomes to DALK in some aspects, it carries a significantly higher risk of complications, as demonstrated by recent meta‐analyses [5, 63, 70]. PK, unlike DALK, is an open‐sky procedure, which poses a higher risk of vision‐threatening intraoperative complications. The incidence of expulsive suprachoroidal hemorrhage is significantly higher in PK, with a reported rate of 0.5%–1.08% [76, 77]. Furthermore, PK carries a threefold higher risk of postoperative endophthalmitis than cataract surgery; DALK minimizes this risk by the closed‐system nature of its approach [78].

The primary advantage of DALK is the preservation of the patient’s own endothelium, preventing progressive endothelial cell loss and eliminating the risk of endothelial graft rejection [79]. In contrast, PK is associated with accelerated endothelial cell loss, which may result from intraoperative trauma caused by instruments, direct contact with the iris or lens, or postoperative biphasic endothelial loss [2, 34]. Other unique complications of PK include trephine‐related injuries to the iris and crystalline lens, as well as penetrating wound issues like wound leakage, iris synechiae, poor wound apposition, and epithelial ingrowth [2, 25].

The Singapore Corneal Transplant Study found significantly higher complication rates in PK than in DALK over a 10‐year follow‐up. PK had higher rates of glaucoma (29.3% vs. 11.6%), epithelial issues (10.4% vs. 5.5%), allograft rejection (16.6% vs. 1.7%), and nonimmunological failure (7.8% vs. 1.9%). Graft failure in PK was mainly due to rejection and endothelial failure (36.7% each), whereas rejection accounted for only 5.9% of failures in DALK [59]. DALK reduces these complications by preserving the patient’s own endothelium, avoiding an open‐sky procedure, and minimizing the risk of cataracts and glaucoma due to a shorter duration of steroid therapy [35, 70, 80]. DALK is also believed to offer a better biomechanical profile, making it less prone to posttraumatic wound dehiscence; this has been further confirmed in a recent meta‐analysis of 11 studies [81].

### 5.1. Complications Specific to DALK

DALK is associated with a distinct set of complications, including DM microperforation leading to a double anterior chamber, DM folds, interface haze, and vascularization [2, 82]. DM perforation is the most frequent intraoperative complication of DALK, even among experienced surgeons, with reported rates ranging from 3% to 37.3% usually leading to conversion to PK [31, 61, 83, 84]. Several techniques have been developed to salvage DALK despite DM perforation, including stromal or amniotic membrane patching, fibrin glue application, and perforation suturing [85–89]. Goweida et al. reported the outcomes of 12 eyes in which DALK was completed despite DM perforations larger than 4 mm, with all DM detachments successfully managed using an intracameral injection of air or SF6 [90]. Inadvertent DM perforation has been linked to early postoperative DM detachment, leading to double AC formation. While spontaneous resolution has been reported, management often requires AC rebubbling with air or gas [91, 92]. However, gas tamponade can lead to endothelial cell loss exceeding 20%, due to high IOP from pupillary block or direct contact with the gas or air [2, 90, 93].

Suture‐related complications, including loosening, vascularization, and sterile infiltrates, are more frequently observed in DALK than PK [94]. Khattak reported a higher incidence of loose sutures in DALK (21.3%) compared to PK (4.0%), requiring re‐suturing in 10.2% of DALK cases and 3.0% of PK cases [70]. Similarly, Feizi et al. found suture complications in 72.0% of DALK cases versus 48.6% in PK, with suture‐related graft rejection occurring in 8.3% and 4.6% of cases, respectively [31]. This higher rate in DALK is likely due to the greater elasticity of younger recipient corneas, increased posterior pressure, and faster graft–host junction healing. Additionally, surgeons may place sutures more superficially in DALK to avoid perforating the thin recipient DM [31, 70].

## 6. Innovations and Modification in DALK Surgery

Unlike endothelial keratoplasty, which has rapidly gained popularity, DALK has struggled to become widely adopted in the United States over the past decade. According to the 2022 Eye Bank Association of America report, PK accounted for 33% of corneal transplants, while DALK remained at 1%. PK was the predominant procedure for corneal ectasia (91.2%), and the number of anterior lamellar keratoplasties has remained largely unchanged over the past 9 years [95].

The main barrier to DALK adoption is the technical challenge of baring the DM to ensure a smooth graft–host interface. Standardization and simplification are essential for broader acceptance. Several modifications and innovations have been developed to address this issue.

### 6.1. BB Technique: Challenges and Modifications

Among the various surgical approaches introduced to achieve lamellar dissection of the anterior corneal stroma, the BB technique, developed by Anwar and Teichmann, remains the most widely adopted method for deep lamellar dissection [6]. In this approach, air is injected into the deep stroma to create a plane of separation between the posterior stroma and either the pre‐Descemetic layer (Type 1 BB) or Descemet’s membrane (Type 2 BB). This pneumatic dissection enables safe and uniform removal of the anterior stroma, providing a smoother graft–host interface [96, 97].

A primary challenge of this approach is achieving optimal cannula depth when accessing the central cornea—ideally within 100 μm of the posterior cornea [98]—since it relies on subjective indicators, increasing the risk of inadvertent DM perforation. Subsequent studies have demonstrated that peripheral air injection outside the trephination site produces outcomes comparable to central corneal injection [99]. Moreover, reaching the central cornea is not essential, as air injection within 1‐2 mm of a deep trephination is sufficient for effective pneumatic dissection [100]. These adaptations can assist surgeons new to this procedure in achieving successful outcomes. Intraoperative optical coherence tomography (OCT) has been employed in DALK to assist in determining trephination depth and visualizing injection needle placement, ensuring optimal depth. This technology enhances procedural accuracy and safety by reducing the risk of DM perforation and minimizing recipient bed folds [101–103].

### 6.2. Femtosecond Laser–Assisted DALK (Femto‐DALK)

Femto‐DALK is an emerging technology that has been used to enhance the DALK procedure in multiple ways. It enables the creation of customized trephination patterns, such as the “mushroom,” “top hat,” or “zig‐zag” profiles, at a specified depth. These configurations improve donor–recipient alignment, create an integrated graft–host junction, and enhance structural integrity, leading to faster wound healing [104–106]. Additionally, Femto‐DALK allows for accurate control of dissection depth, and deeper cannula placement may increase the likelihood of BB formation. Laser‐created stromal channels further facilitate posterior lamellar plane dissection, reducing the risk of DM perforation (25.9% vs. 45.4% in conventional DALK) and conversion to PK (3.4% vs. 24.5%) [107]. A recent meta‐analysis by Du et al., which included nine controlled studies involving 1713 eyes, compared the efficacy and safety of Femto‐DALK and conventional DALK in the management of keratoconus. The findings indicated that Femto‐DALK provides superior early visual acuity and a lower intraoperative DM perforation rate [108]. Gonzales et al. also reported a lower risk of graft rejection at 1 year with Femto‐DALK (9.3%) compared to conventional DALK (20.2%), probably due to increased scar formation at the host–graft junction, which prohibits immune cells from reaching the donor stroma [32]. Finally, femtosecond laser–assisted astigmatic keratotomy (FSAK) is a safe and effective technique to address postkeratoplasty astigmatism [109, 110].

### 6.3. Large‐Diameter DALK

Some studies suggest that large‐diameter grafts in DALK may yield superior visual outcomes, with reduced myopia and astigmatism [111, 112]. Lucisano et al. compared the clinical outcomes of large‐diameter (9.0 mm) and conventional (8.0 mm) BB DALK. The proportion of eyes achieving visual acuity of 20/20 or better was significantly higher in the 9.0‐mm DALK group (44%) compared to the 8.0‐mm DALK group (26%). Refractive astigmatism was also significantly lower in the 9.0‐mm DALK group at all postoperative time points. Meanwhile, postoperative complication rates were comparable between the two groups [113]. Notably, in contrast to PK, large‐diameter DALK does not increase the risk of immune‐mediated stromal rejection [114, 115].

### 6.4. Role of Artificial Intelligence (AI) in Predicting Outcomes

The integration of AI in ophthalmology is rapidly gaining popularity. AI models can analyze OCT images and patient data to optimize BB formation in DALK and predict graft survival or rejection [116]. Feizi et al. employed five machine learning models to identify variables predictive of graft rejection. According to these algorithms, key predictors of corneal graft rejection in keratoconus patients included short course of corticosteroids (4 months), PK, prolonged suture retention, and suture‐related complications [117]. In another study, Ling et al. utilized a combination of machine learning and traditional statistical methods to identify predictors of long‐term graft survival following PK and DALK, using data from the Singapore Corneal Transplant Registry. They identified five key factors as predictors of 10‐year graft survival: recipient sex (with males at higher risk), preoperative visual acuity, choice of procedure, surgical indication, and active inflammation [118].

## 7. Broader Clinical and Global Perspectives

### 7.1. Quality of Life and Patient Satisfaction

Although the primary goal of DALK is to improve visual acuity, studies have shown that it also leads to a significant improvement in vision‐related quality of life, which continues to increase after suture removal [119, 120].

In terms of patient satisfaction, both PK and DALK achieved similarly high and satisfactory outcomes. However, 84.6% of patients who underwent DALK and 76% of those who underwent PK indicated that they would choose to have the surgery again, showing slightly higher satisfaction among DALK patients [35].

### 7.2. Tissue Accessibility and Cost‐Effectiveness

Despite an estimated 8 million people worldwide living due to corneal blindness, the number of corneal transplants performed annually remains insufficient to meet this need [121, 122]. Many countries face persistent shortages due to high demand, limited donor availability, and underdeveloped eye banking infrastructure [123]. In this context, DALK offers a valuable opportunity to enhance donor tissue utilization.

DALK selectively replaces only the diseased anterior layers of the cornea while preserving the healthy posterior layers. This approach allows the use of donor corneas with suboptimal endothelial conditions that would not be appropriate for PK. Moreover, DALK offers improved long‐term graft survival, thereby reducing the likelihood of regrafting [34]. An additional advantage is its role in dual‐purpose tissue use, enabling a single donor cornea to serve two recipients, one for DALK and another for Descemet membrane endothelial keratoplasty (DMEK) [124]. Collectively, these factors contribute to a meaningful expansion of the effective donor pool [125].

Considering the constrained resources of healthcare systems worldwide, cost‐effectiveness evidence can play a pivotal role in guiding both clinicians and policymakers toward optimal decision‐making [126]. The cost‐effectiveness of DALK compared with PK for keratoconus was evaluated over 20 years at the Singapore National Eye Centre, showing that although both procedures are highly cost‐effective, DALK exhibited a lower cost‐utility ratio, indicating greater cost‐effectiveness and supporting its consideration as the preferred approach [127]. Similarly, a randomized multicenter trial in the Netherlands found DALK to be more effective than PK, with its cost‐effectiveness likely to improve over time due to higher graft survival rates [128].

To provide a concise overview of the comparative evidence, Table 2 highlights the key distinctions between DALK and PK with respect to graft survival, endothelial health, visual and refractive outcomes, immune response, complications, and cost‐effectiveness.

**TABLE 2:** Comparison between DALK and PK in terms of clinical and surgical outcomes.

	DALK	PK	Key remarks
Graft survival	Superior long‐term graft survival (> 90% at 10 years) due to absence of endothelial rejection	Lower long‐term survival due to endothelial rejection and progressive cell loss	DALK eliminates risk of endothelial rejection and offers significantly better graft longevity
ECD & ECL	Minimal postoperative ECD loss (typically 10%–20%)	Marked postoperative ECD loss (25%–50%)	DALK preserves host endothelium, maintaining long‐term function
Visual outcomes	Comparable CDVA to PK when a successful big‐bubble and thin residual stromal bed are achieved	Good CDVA but dependent on graft clarity and endothelial health	With optimal technique, DALK achieves visual outcomes equivalent to PK
Refractive outcomes	Slightly higher mean myopia (∼1 D more) and comparable astigmatism	Slightly lower spherical equivalent and corneal curvature	Differences are statistically minor and not clinically significant; both yield stable long‐term refraction
Immune rejection	No endothelial rejection; stromal rejection is possible but responds well to treatment	Significant risk of endothelial rejection and subsequent graft failure	Major long‐term advantage of DALK in maintaining graft clarity
Complications	Intraoperative DM perforation (5%–15%), interface haze, double anterior chamber	Endothelial rejection, secondary glaucoma, late endothelial failure	DALK is safer in the long term and avoids endothelial rejection
Tissue Accessibility	Can utilize corneas with suboptimal endothelial quality; enables split‐cornea use (e.g., DALK + DMEK)	Requires donor tissue with excellent endothelial quality	DALK expands donor pool and optimizes tissue use
Cost‐effectiveness	More cost‐effective long term due to fewer re‐grafts and better graft survival	Cost‐effective but less durable	Both are cost‐effective, but DALK is considered the preferred surgical approach

Abbreviations: CDVA, corrected distance visual acuity; DALK, deep anterior lamellar keratoplasty; DMEK, Descemet membrane endothelial keratoplasty; ECD, endothelial cell density; ECL, endothelial cell loss; PK, penetrating keratoplasty.

## 8. Conclusions

Graft survival is the primary measure of keratoplasty success. Long‐term studies (> 10 years) have shown superior graft survival and higher ECDs with DALK compared to PK. The key advantage of DALK over PK lies in preserving the patient’s endothelium, thereby eliminating the risk of endothelial immune rejection—the leading cause of graft failure. Furthermore, DALK offers a safer intraoperative profile, with reduced complications and lower endothelial cell loss. Despite both procedures achieving comparable visual acuity outcomes, these benefits make DALK the preferred choice whenever feasible.

Another key benefit of DALK is its independence from donor endothelial quality, making it particularly valuable in regions with limited access to human donor corneas. This also expands the donor pool, as corneas unsuitable for PK due to poor endothelial viability can still be successfully used for DALK. Recent advancements in surgical techniques have improved the standardization and success rates of DALK, thereby increasing its adoption. Future research should focus on addressing existing gaps, such as conducting randomized trials to compare emerging surgical modifications and validating the role of AI models in predicting outcomes.

In summary, DALK should be considered the first‐line surgical option for patients with corneal stromal diseases with healthy endothelium.

## Funding

No funding was received for this manuscript.

## Conflicts of Interest

The authors declare no conflicts of interest.

## Data Availability

Data sharing is not applicable to this article as no datasets were generated or analyzed during the current study.
